# NECTIN2 depletion impairs migration and invasion in MDA-MB-231 breast cancer cells through LIMK1-associated cytoskeletal remodeling

**DOI:** 10.1371/journal.pone.0356726

**Published:** 2026-08-28

**Authors:** Phatchanat Klaihmon, Pattaratorn Muangtate, Sudarat Thongphayong, Puretat Saetan, Supasorn Chanthateyanonth, Chanitra Thuwajit, Surapol Issargrisil, Phatchariya Phannasil

**Affiliations:** 1 Siriraj Center of Excellence for Stem Cell Research, Faculty of Medicine Siriraj Hospital, Mahidol University, Bangkok, Thailand; 2 Molecular Cancer Therapeutics Research Group, Faculty of Medicine Siriraj Hospital, Mahidol University, Bangkok, Thailand; 3 Master of Science Program in Molecular Genetics and Genetic Engineering, Institute of Molecular Biosciences, Mahidol University, Nakhon Pathom, Thailand; 4 Thalassemia Research Center, Institute of Molecular Biosciences, Mahidol University, Nakhon Pathom, Thailand; 5 Doctor of Philosophy Program in Systems Biosciences, Institute of Molecular Biosciences, Mahidol University, Nakhon Pathom, Thailand; 6 Department of Immunology, Faculty of Medicine Siriraj Hospital, Mahidol University, Bangkok, Thailand; Mayo Clinic Cancer Center, UNITED STATES OF AMERICA

## Abstract

Breast cancer (BC) is the most commonly diagnosed malignancy in women, with triple-negative breast cancer (TNBC) representing the most aggressive subtype that is associated with poor clinical outcomes. Identifying novel biomarkers or therapeutic targets is therefore of great importance. Nectin cell adhesion molecule-2 (NECTIN2) is an immunoglobulin-like glycoprotein involved in cell adhesion and immune regulation that is highly expressed in several cancers but its role in the progression of BC is unclear. Using publicly available data sets, we found that NECTIN2 expression is elevated in BC compared to normal tissues and is associated with poorer recurrence-free survival in TNBC patients. Among TNBC cell lines, MDA-MB-231 cells exhibited the highest expression of NECTIN2 at gene and protein levels, consistent with a more aggressive phenotype. To investigate its functional role, we silenced NECTIN2 in MDA-MB-231 and MDA-MB-468 cells. Relative cell viability was modestly reduced in NECTIN2-depleted MDA-MB-231 cells at later time points, but no significant changes were observed in MDA-MB-468 cells. Interestingly, NECTIN2 depletion significantly reduced migration and invasion in MDA-MB-231 cells, but not in MDA-MB-468 cells. Given this cell line–specific effect, subsequent mechanistic investigations focused on the MDA-MB-231 model. Gene expression profiling suggested a reduction in LIMK1, a regulator of cytoskeletal dynamics and cell motility, following NECTIN2 depletion. Furthermore, ectopic expression of LIMK1 in NECTIN2-deficient MDA-MB-231 cells partially restored migratory capacity and was associated with partial epithelial–mesenchymal transition (EMT)-like changes, including a decrease in E-cadherin and ZO-1, and a higher level of fibronectin and Slug. Collectively, these findings suggest that NECTIN2 contributes to migratory and invasive phenotypes in MDA-MB-231 cells, potentially through LIMK1-associated cytoskeletal remodeling and EMT-related marker changes. However, this mechanism requires further investigation because it appears to be context-dependent and may not be universally applicable across TNBC subtypes.

## Introduction

Breast cancer (BC) is the most commonly diagnosed malignancy and the leading cause of cancer-related death in women, with 2.3 million new cases and 665,684 deaths reported in 2022 [1]. BC is broadly classified into four major molecular subtypes—Luminal A, Luminal B, human epidermal growth factor receptor 2-enriched (HER2-enriched), and triple-negative breast cancer (TNBC)—based on the expression of estrogen receptor (ER), progesterone receptor (PR), and HER2, which guide therapeutic strategies [2–4]. Among these subtypes, TNBC is recognized as the most aggressive form with its rapid progression [5] and significantly poorer survival outcomes compared to other subtypes of breast cancer [6]. Once distant metastasis develops, the prognosis remains poor, with a median overall survival of approximately 12–18 months despite systemic therapy [4], underscoring the need to identify novel biomarkers and therapeutic targets for TNBC.

Nectin cell adhesion molecule-2 (NECTIN2) is a Ca^2+^-independent immunoglobulin-like glycoprotein that plays an important role in the regulation of immune cells and control of the cell-to-cell adhesive process during cancer progression [7]. High levels of NECTIN2 protein have been observed in many types of cancers and could be used as a prognostic biomarker for colorectal carcinoma [8], pancreatic ductal adenocarcinoma [9], esophageal squamous cell carcinoma [10] and lung cancer [11]. Given its potential as a therapeutic target, a monoclonal antibody against NECTIN2 was shown to inhibit the growth of ovarian and breast cancer in mouse models, primarily through antibody-dependent cellular cytotoxicity (ADCC) [12]. Similarly, an antibody-drug conjugate directed against NECTIN2 significantly suppressed the progression of NECTIN2-positive ovarian cancer in xenograft models [13].

LIMK1, a serine/threonine kinase that regulates actin‑cytoskeleton dynamics and cell migration, is frequently overexpressed in multiple cancers, including breast cancer, where its cytoplasmic and nuclear forms promote tumor growth and metastasis in vitro and in xenograft models. [14]. The PAK4/LIMK1/Cofilin-1 pathway is upregulated in osteosarcoma, where PAK4 regulates cofilin-1 through LIMK1 to promote proliferation, migration and invasion; inhibition of PAK4 suppresses this pathway and induces apoptosis, while overexpression of LIMK1 rescues these effects [15]. Collectively, LIMK1 acts as a key downstream effector of adhesion molecules and signaling pathways and may mediate the pro‑metastatic effects of NECTIN2 through cytoskeletal remodeling and increased cell motility.

Although NECTIN2 is recognized as a potential therapeutic target, particularly in the context of antibody-based therapies, the functional role of NECTIN2 in regulating cell migration and invasion in TNBC remains unclear. In the present study, we aimed to investigate the role of NECTIN2 in TNBC cell motility using short hairpin RNA (shRNA)-mediated knockdown. Based on the observed phenotypic effects, subsequent mechanistic analyses were primarily performed in MDA-MB-231 cells. Furthermore, we examined whether re-expression of LIMK1 could rescue the effects of NECTIN2 depletion, providing insights into potential LIMK1-associated downstream signaling pathways.

## Materials and methods

### Data mining and database analyses

The mRNA expression levels of *NECTIN2* in normal and tumor tissues were analyzed using the online-available GEPIA2 database (https://gepia2.cancer-pku.cn/). The recurrence-free survival (RFS) curve was generated using the Kaplan-Meier plotter (https://kmplot.com/analysis/) with the JetSet best probe for 203149_at dataset. The correlation analysis between *NECTIN2* and *LIMK1* was performed using GEPIA2 (http://gepia2.cancer-pku.cn/).

### Cell culture

Breast cancer cell lines (MDA-MB-231, MDA-MB-468, and MCF-7) and HEK293FT cells were cultured in high-glucose DMEM supplemented with 10% FBS, 10 μM GlutaMAX, and antibiotics (100 U/mL penicillin and 100 μg/mL streptomycin) at 37 °C in 5% CO_2_. MDA-MB-231 and MCF-7 cells were kindly provided by Assoc. Prof. Dr. Chareeporn Akekawatchai (Thammasat University), MDA-MB-468 cells by Assoc. Prof. Dr. Supannikar Tawinwung (Chulalongkorn University), and HEK293FT cells were purchased from Thermo Fisher Scientific (Carlsbad, CA, USA). All cell lines were tested and confirmed to be free of mycoplasma before use.

### Generation of *NECTIN2*-KD breast cancer cells

shRNA targeting human *NECTIN2* (#sc-43169-SH; Santa Cruz Biotechnology) and scramble shRNA (#VB190408-1223usc; VectorBuilder) were used to generate lentiviral particles in HEK293FT cells together with pCMV.dR8.2 dvpr and pCMV-VSV-G plasmids (Addgene #8454 and #8455) in a 5:4:1 ratio using Lipofectamine 3000 (Thermo Fisher Scientific). After 48 hours, the particles were harvested and concentrated using Amicon Ultra-15 centrifugal filters (Merck Millipore, Tullagreen, Ireland) at 4,500 × g for 1 h.

A total of 5 × 10^5^ cells MDA-MB-231 and MDA-MB-468 were transduced with concentrated viral particles in the presence of 8 μg/mL of polybrene (Miltenyi Biotec). After medium replacement at 24 h, cells were cultured for 48 h and selected with puromycin (2 μg/mL), and stable cells were maintained in 0.5 μg/mL puromycin.

### Flow cytometric analysis

For detection of cell surface proteins, 1x 10^5^ breast cancer cells were incubated with PE-conjugated anti-human NECTIN2 (Biolegend) in a FACS buffer for 15 min at room temperature in the dark. Then, stained samples were washed twice with cold PBS and subjected to FACS analysis (FACS Canto, BD Biosciences, USA).

### MTT assay

Cell proliferation was evaluated by an MTT assay in wild-type (WT), scramble (SCR), and *NECTIN2* knockdown (KD) MDA-MB-231 and MDA-MB-468 cells. Cells were seeded at 5,000 cells/well in 96-well plates and incubated overnight. MTT was added to a final concentration of 0.5 mg/mL and incubated for 1 h, followed by dissolution of formazan crystals in DMSO. Absorbance at 570 nm was measured to assess cell viability. The assay was performed for three consecutive days and proliferation was calculated by normalizing OD values to day 0.

### RT-qPCR analysis

Total RNA was extracted from breast cancer cells using TRIzol reagent (Thermo Fisher Scientific, USA). cDNA was synthesized from 250 ng of RNA using oligo(dT)₁₈ primers and the RevertAid First Strand cDNA Synthesis Kit (Thermo Fisher Scientific, USA) according to the manufacturer’s instructions. Quantitative PCR was performed using 2 × iTaq Universal SYBR Green Supermix (Bio-Rad) with gene-specific primers (listed in S1 Table). The PCR conditions were 95 °C for 2 min, followed by 40 cycles of 95 °C for 15 s and gene-specific annealing/extension for 1 min. Gene expression was normalized to β-actin and calculated using the 2^−ΔCT^ method.

### Ectopic expression of LIMK1

The LIMK1 plasmid (#SC1200, cloned into a pcDNA3.1 + /C-(K)-DYK vector backbone) was purchased from GenScript (Piscataway, NJ, USA). 2 x 10^6^ MDA-MB-231 cells were mixed with 5 μg plasmid DNA in SE Cell Line Nucleofector™ solution (Lonza, Basel, Switzerland), and nucleofected using the 4D-Nucleofector™ system with the CA-137 program. Following nucleofection cells were transferred to pre-warmed complete medium, then allowed to recover and reach subconfluence prior to use in downstream rescue experiments.

### LIMK1 depletion

To investigate the functional role of LIMK1 in cancer cell invasiveness, CRISPR/Cas9-mediated gene depletion was performed. MDA-MB-231 cells were transduced for 24 hours with concentrated lentiviral particles containing the gRNA plasmid targeting LIMK1 (#76047, Addgene). Lentiviral particles were pre-produced in HEK293FT cells by co-transfecting the gRNA plasmid alongside packaging plasmids dR8.2 and VSV-G using Lipofectamine reagent. Following transduction, stably depleted cells were selected using puromycin at a concentration of 2 µg/mL for 3 days. Parental, non-transduced MDA-MB-231 cells maintained in parallel without puromycin served as the control group. LIMK1 depletion was confirmed by western blotting prior to migration and invasion assays.

### Western blotting analysis

Cells were pelleted and lysed using RIPA buffer (Cell Signaling Technologies). The cell lysates were clarified by centrifugation and protein concentration was quantified using a BCA assay (Pierce Biotechnolgy, Rockford, IL, USA). Equal amounts of protein were resolved in SDS-PAGE under reducing conditions and transferred to a PVDF membrane. Next, the membrane was incubated with anti-NECTIN2 (#95333), anti-LIMK1 (#3842), anti-E-cadherin (#3195), anti-ZO-1 (#8193), anti-Slug (#9585), anti-Fibronectin (#26836), anti-cofilin (#5175) and anti-phospho-cofilin (#3313) primary antibodies (1:1,000, Cell Signaling Technologies, MA, USA). Β-actin was used as a loading control to ensure equal protein loading. This was followed by an HRP-conjugated anti-rabbit IgG secondary antibody (1:5,000, Sigma Aldrich, St Louis, MA). The immune complex was detected by the Immobilon® Western Chemiluminescent HRP substrate (Millipore).

### Wound healing assay

The ability of MDA-MB-231 and MDA-MB-468 cells to migrate was evaluated using a wound healing assay. Cells were seeded at 1.5 × 10^5^ cells/well (MDA-MB-231) or 2.0 × 10^5^ cells/well (MDA-MB-468) in 24-well plates to reach 80–90% confluency, and an artificial wound was created using a 200 µL pipette tip. After washing with PBS, cells were incubated in serum-free DMEM. Wound closure was monitored at 0, 24, and 48 h under 100 × magnification. Images from five random fields per well were captured, and wound area was quantified to calculate gap closure relative to 0 h.

### Transwell Migration and Invasion Assay

Cell migration and invasion assays were performed using Transwell inserts (6.5-mm diameter polyvinylpyrrolidone-free polycarbonate membranes with 8-µm pores; Corning, USA). For the migration assay, 5 × 10⁴ MDA-MB-231 or MDA-MB-468 cells were resuspended in 200 µL of serum-free DMEM and seeded in the upper chamber, while 600 µL of DMEM containing 10% (v/v) FBS (Gibco; Thermo Fisher Scientific, USA) was added to the lower chamber as a chemoattractant. After incubation at 37°C for 24 h, the migrated cells adhering to the lower membrane surface were fixed with 4% paraformaldehyde in 1 × PBS for 20 min at room temperature and stained with 0.5% crystal violet in 25% methanol for 1 h. For the invasion assay, the same number of cells was seeded into Matrigel-coated inserts (30 µg; BD Biosciences) under otherwise identical conditions. Migrated and invaded cells were counted in five randomly selected fields under a light microscope, and the results were plotted as bar graphs.

### Immunofluorescent staining

Pre-confluent MDA-MB-231 cells in a 24-well plate were fixed with 4% paraformaldehyde, blocked with 3% bovine serum albumin, and incubated with anti-LIMK1 primary antibody overnight at 4^o^C. Next, the cells were washed three times with PBS, followed by incubation with Alexa Fluor 594–conjugated goat anti-rabbit IgG secondary antibody. F-actin was stained with Alexa Fluor 488–conjugated phalloidin, and nuclei were counterstained with DAPI/Anti-fade solution (Merck KGaA, Darmstadt, Germany). Images were taken under a fluorescent microscope (Eclipse Ti-U with Nis-Elements, Nikon).

### Statistical analysis

The results were obtained from three independent experiments performed in duplicate and are presented as mean ± SEM. Statistical analyzes were conducted using the unpaired Student’s t-test, or one-way ANOVA followed by Tukey’s post hoc test with GraphPad Prism version 10.0. A p-value < 0.05 was considered statistically significant. For experiments performed using technical replicates, the replicate values were averaged before statistical analysis.

## Results

### Expression of NECTIN2 in breast cancer cells

**Fig 1:**
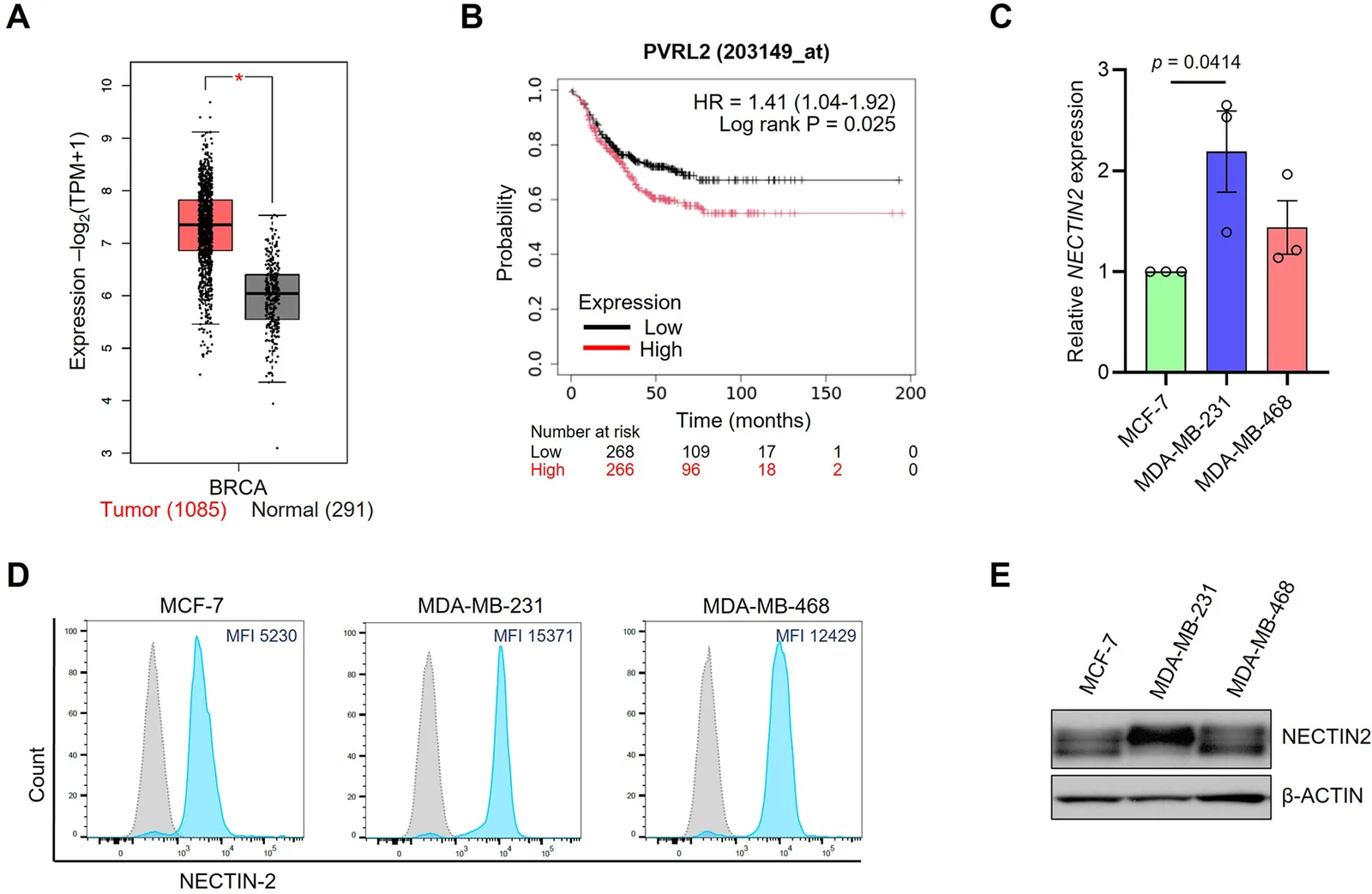
NECTIN2 expression is elevated in breast cancer and is highly expressed in TNBC cells. (A) Analysis of the GEPIA2 database revealed that NECTIN2 mRNA expression was significantly upregulated in breast cancer tissues (BRCA) compared with normal tissues. (B) High NECTIN2 expression was associated with recurrence-free survival (RFS) in TNBC patients. (C) qRT-PCR, (D) flow cytometry, and (E) western blot analyzes showed that MDA-MB-231 cells expressed markedly higher levels of NECTIN2 mRNA and protein compared to MDA-MB-468 and MCF7 cells, indicating that NECTIN2 expression correlates with the aggressive TNBC phenotype. Data are presented as mean ± SEM. Statistical significance was determined using one-way ANOVA followed by Tukey’s multiple comparisons test. n = 3 independent biological replicates, each performed in duplicate technical replicates.

Using the available GEPIA2 database, the expression of *NECTIN2* in breast cancer (BRCA) patients was significantly upregulated compared to normal tissues (Fig 1A). We identified a significant association with recurrence-free survival (RFS) (HR = 1.41, 95% CI = 1.04-1.92, *p* = 0.025). High expression of *NECTIN2* was correlated with poorer RFS in TNBC patients (Fig 1B). Moreover, we assessed the NECTIN2 expression levels at both the gene and protein levels in TNBC cell lines, including MDA-MB-231 cells, MDA-MB-468 cells, and luminal A subtype breast cancer MCF7 cells. At the gene level, quantitative RT-PCR analysis revealed that MDA-MB-231 cells exhibited the highest expression of NECTIN2, with a 2-fold increase compared to MDA-MB-468 cells and MCF7 cells (p < 0.05) (Fig 1C). At the protein level, the flow cytometry data further corroborated these findings, with MDA-MB-231 cells displaying higher NECTIN2 protein expression compared to MDA-MB-468 and MCF7 cells, respectively (Fig 1D). Western blot analysis also confirmed that MDA-MB-231 cells had the highest NECTIN2 protein levels, showing a 2-fold increase in protein expression relative to MDA-MB-468 cells and MCF7 cells (p < 0.05) (Fig 1E). These results demonstrate a clear correlation between the NECTIN2 expression levels at both the gene and protein levels across different methods, confirming that MDA-MB-231 cells have the highest NECTIN2 expression. This data suggests that NECTIN2 expression may be associated with aggressiveness of TNBC type.

### NECTIN2 depletion leads to a delayed reduction in cell viability in MDA-MB-231 cells

**Fig 2:**
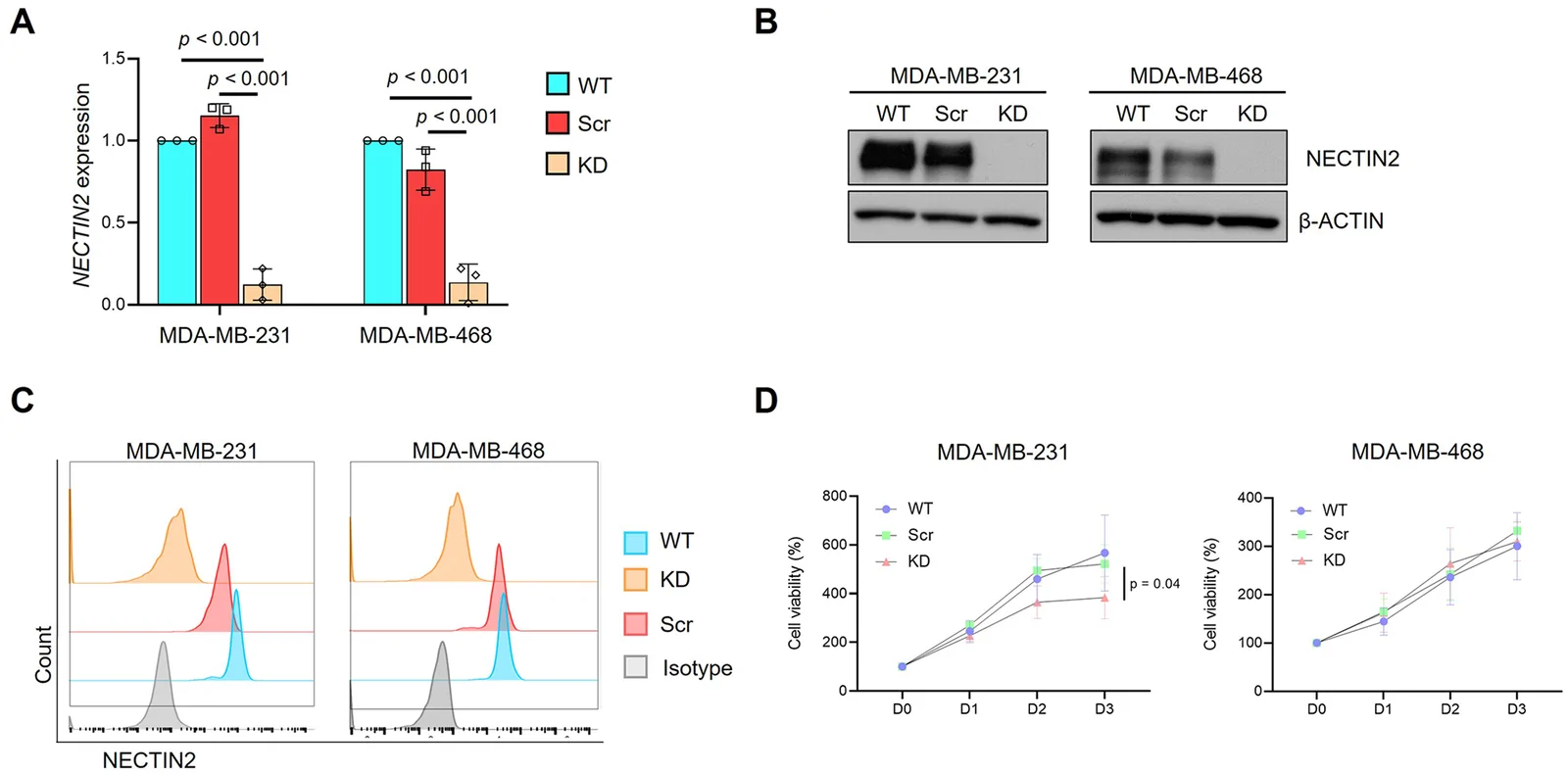
NECTIN2 depletion modestly reduces viability in MDA-MB-231 cells. (A) RT-qPCR (B) Western blot, and (C) flow cytometry analyzes showing efficient NECTIN2 knockdown in MDA-MB-231 and MDA-MB-468 cells compared to SCR and WT cells. (D) Relative cell viability, measured by MTT assay over three days and normalized to Day 0, showed a significant reduction in NECTIN2-depleted MDA-MB-231 cells at Day 3 compared to WT and SCR controls, while no significant differences were observed in MDA-MB-468 cells. Data are presented as mean ± SEM. Statistical significance was determined using one-way ANOVA followed by Tukey’s multiple comparison test. n = 3 independent biological replicates, each performed with 2- to 3 technical replicate wells per condition.

To decipher the role of NECTIN2 in breast cancer cells, we utilized shRNA to reduce the expression of NECTIN2 in TNBC breast cancer cell lines, including MDA-MB-231 and MDA-MB-468 that exhibit the highest and moderate levels of NECTIN2 expression, respectively. Quantitative RT-PCR, Western blot and flow cytometry analyses were performed to confirm the successful downregulation of NECTIN2 in these TNBC cell lines. Specifically, RT-qPCR revealed a significant reduction in NECTIN2 expression by approximately 90% in both MDA-MB-231 and MDA-MB-468 cells (p < 0.05) (Fig 2A). Western blot analysis showed a significant reduction of NECTIN2 protein levels in both MDA-MB-231 and MDA-MB-468 cells compared to scramble control and their parental cells (Fig 2B). Flow cytometric analysis further confirmed the downregulation of NECTIN2 at the cell surface in both cell lines (Fig 2C).

Relative cell viability, assessed by the MTT assay over three consecutive days and normalized to Day 0, showed a significant reduction in NECTIN2-knockdown (KD) MDA-MB-231 cells at Day 3 compared to WT and SCR controls. Notably, this difference became apparent only at the later time point, as no significant changes were observed at earlier time points. In contrast, no significant changes were observed in MDA-MB-468 cells. These results suggest that NECTIN2 downregulation modestly reduces cell growth/viability in MDA-MB-231 cells, but not in MDA-MB-468 cells (Fig 2D).

### Depletion of NECTIN2 decreased TNBC cell migration and invasion

**Fig 3:**
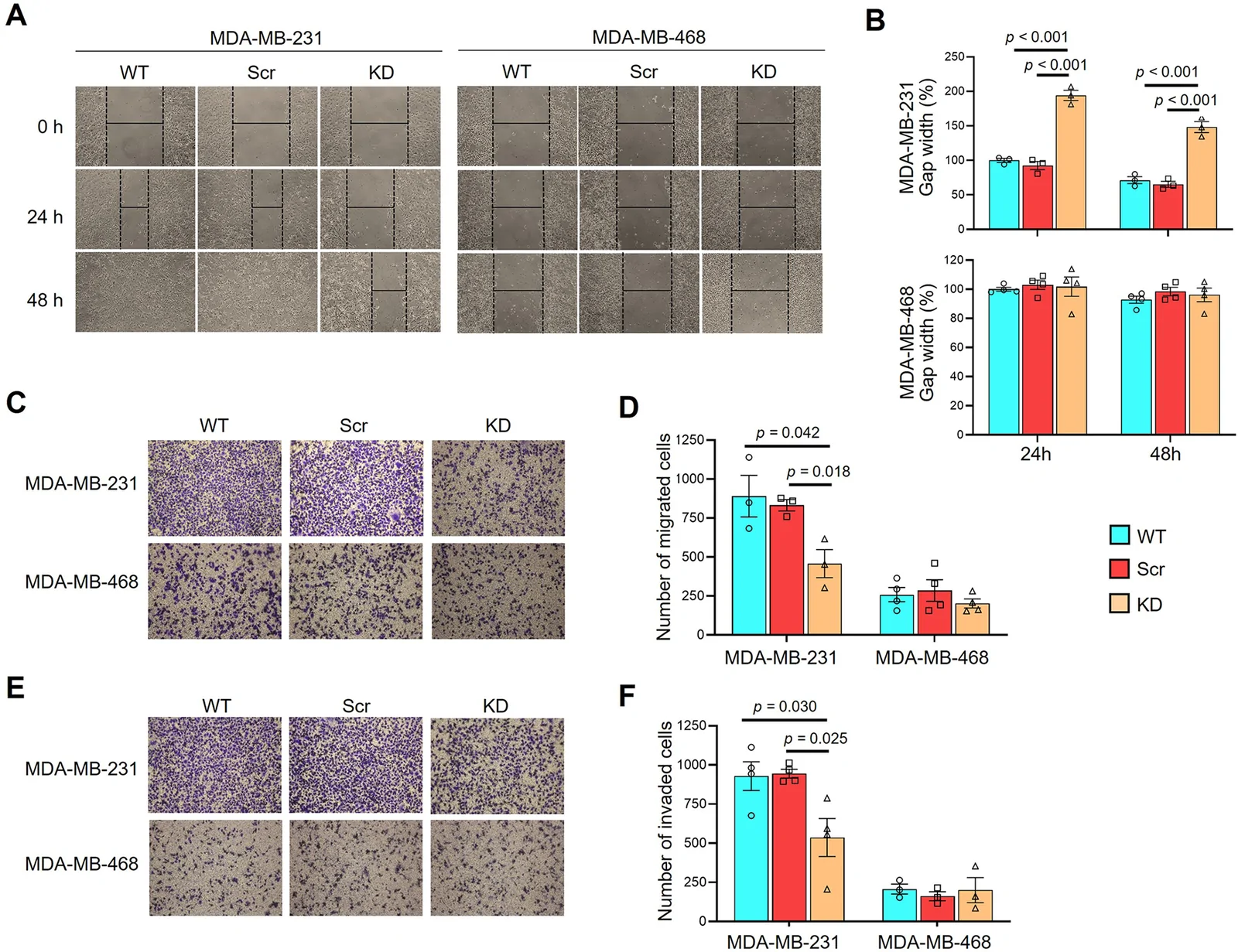
NECTIN2 depletion impairs migration and invasion of MDA-MB-231 TNBC cells. (A) Representative wound healing images and (B) quantification of gap width showing delayed wound closure in NECTIN2-KD MDA-MB-231 cells compared with WT and SCR controls. (C) Representative Transwell migration images of TNBC cells after NECTIN2 knockdown. (D) The number of migrated cells showing a significant decrease in NECTIN2-KD MDA-MB-231 cells compared with WT and SCR controls. (E) Representative Transwell invasion images showing the reduced invasive ability of NECTIN2- KD MDA-MB-231 cells. (F) The number of cells that show a reduction in invasion after NECTIN2 knockdown, while no significant change was detected in MDA-MB-468 cells. Data are presented as mean ± SEM. Statistical significance was determined using one-way ANOVA followed by Tukey’s multiple comparisons test. n = 3 independent biological replicates, each performed in duplicate technical replicates.

Because NECTIN2 is considered one of the cell adhesion molecules, we investigated the role of NECTIN2 on TNBC migration and invasion. The depletion of NECTIN2 in TNBC cells led to a significant impairment of cancer cell migration, as assessed by both scratch-wound and Transwell assays. In the scratch-wound assay, NECTIN2 knockdown in MDA-MB-231 cells reduced wound closure by approximately 50% at both 24 and 48 h compared to WT and SCR control cells (Fig 3A–B), indicating a substantial decrease in migratory capacity. No significant change was observed in MDA-MB-468 cells.

Similarly, in the Transwell migration assay, NECTIN2 knockdown in MDA-MB-231 cells resulted in a 50% reduction in the number of cells migrating through the membrane compared to WT and SCR controls (Fig 3C–D), confirming impaired migration. To evaluate the effect of NECTIN2 depletion on invasive potential, a Transwell invasion assay was performed. In MDA-MB-231 cells, NECTIN2 knockdown reduced the number of invasive cells by approximately 50% relative to WT and SCR controls. In contrast, no significant reduction in migration or invasion was observed in MDA-MB-468 cells, suggesting that the impact of NECTIN2 depletion on metastatic behavior is specific to MDA-MB-231 cells (Fig 3E–F).

### Identification of downstream target affected by suppression of NECTIN2

Given the marked reduction in migration and invasion observed in *NECTIN2*-knockdown MDA-MB-231 cells, but not MDA-MB-468 cells, we proceeded to elucidate the molecular mechanisms underlying this cell line specific phenotype. Therefore, all subsequent mechanistic analyzes were focused on MDA-MB-231 cells.

**Fig 4:**
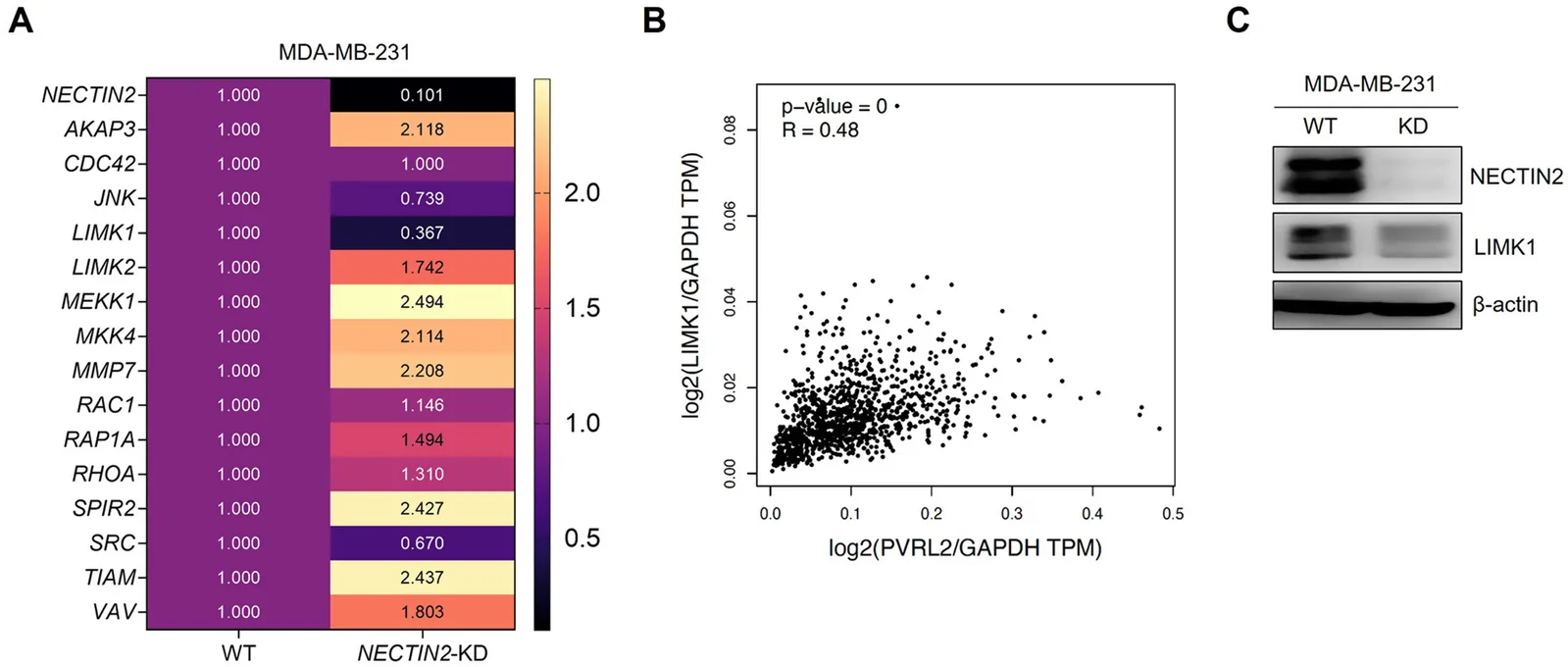
Identification of LIMK1 as a downstream target of NECTIN2 in MDA-MB-231 cells. (A) RT-qPCR analysis of 15 candidate genes potentially regulated by NECTIN2. LIMK1, a key regulator of actin cytoskeletal dynamics, was downregulated after NECTIN2 knockdown. (B) Correlation analysis of *NECTIN2 (PVRL2)* and *LIMK1* expression using the GEPIA2 database in breast cancer datasets revealed a moderate positive and statistically significant correlation (R = 0.48, p < 0.0001). (C) Western blot analysis that confirms that LIMK1 protein levels were markedly reduced in NECTIN2-depleted MDA-MB-231 cells compared to WT controls. Expression data represent the mean of two independent biological replicates, each performed in duplicate technical replicates. Statistical analysis was performed using an unpaired Student’s t-test.

To identify signaling pathways potentially regulated by NECTIN2 during migration and invasion, we performed RT-qPCR to quantify the expression of 15 candidate genes. These genes were selected based on their established roles in cytoskeletal regulation, implicated in cell motility and invasion, as well as evidence from previous studies linking these pathways to NECTIN2-associated signaling [16–19]. The selected genes include *AKAP3, CDC42, JNK, LIMK1, LIMK2, MEKK1, MKK4, MMP7, RAC1, RAP1A, RhoA, SPIR2, SRC, TIAM1* and *VAV*. All expression data represent the mean of two independent biological replicates. Our results revealed distinct transcriptional changes upon NECTIN2 depletion. In particular, key regulators of actin cytoskeletal dynamics and pro-migratory signaling, LIMK1, JNK, and SRC*,* were downregulated in NECTIN2-KD cells. Among them, *LIMK1* showed the strongest reduction (~70%), while JNK and SRC decreased by ~30–40% compared with WT controls. In contrast, several genes were upregulated in NECTIN2-KD cells, including *AKAP3*, *LIMK2, MEKK1, MKK4, MMP7, SPIR2, TIAM1*, and *VAV* (Fig 4A). These changes suggest that NECTIN2 may promote migration and invasion in MDA-MB-231 cells through coordinated regulation of cytoskeletal- and MAPK-related pathways.

To complement our experimental findings, we examined the relationship between *NECTIN2 (PVRL2)* and *LIMK1* expression using the GEPIA2 database. The analysis of data sets derived from breast cancer patients revealed a modest positive correlation (R = 0.48, p < 0.0001) (Fig 4B). Although this association is statistically significant, it represents a moderate positive correlation and may reflect a biologically relevant relationship between NECTIN2 and LIMK1. These findings support the possibility that NECTIN2 and LIMK1 are functionally linked within broader regulatory networks associated with breast cancer. Importantly, our RT-qPCR results in MDA-MB-231 cells showed a robust reduction in LIMK1 after NECTIN2 knockdown, indicating a stronger, cell line–specific regulatory relationship that is not fully captured in the bulk tumor datasets.

To further validate these findings at the protein level, we performed a Western blot analysis to assess LIMK1 expression following NECTIN2 knockdown. Consistent with the RT-qPCR results, NECTIN2-depleted MDA-MB-231 cells exhibited markedly reduced LIMK1 protein levels compared to WT controls (Fig 4C), suggesting the correlation between NECTIN2 and LIMK1 to promote migration and invasion in MDA-MB-231 cells.

### LIMK1 overexpression restores migration in NECTIN2-depleted MDA-MB-231 cells

**Fig 5:**
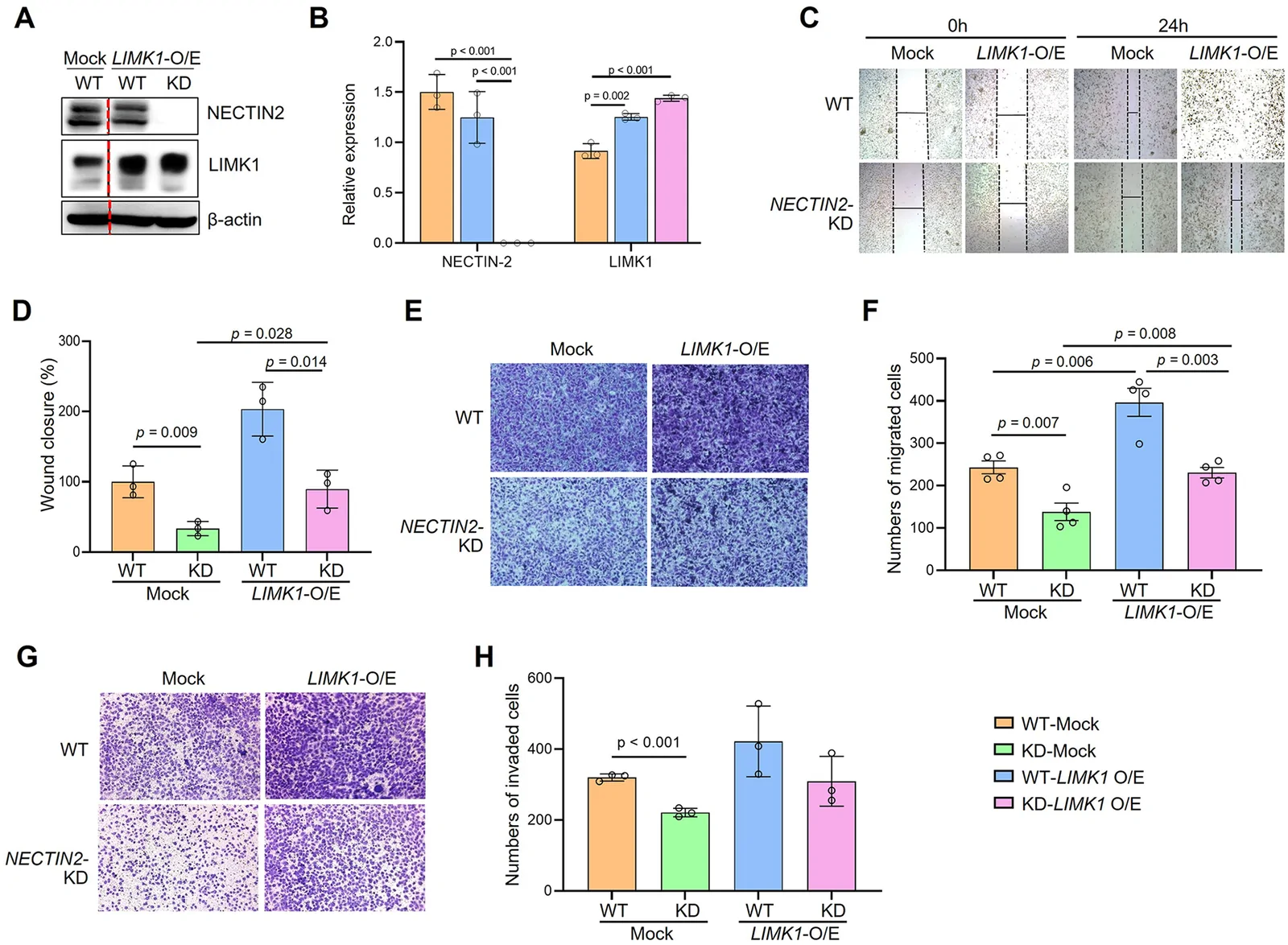
LIMK1 overexpression partially restores the migratory ability of NECTIN2-depleted MDA-MB-231 cells. (A) Western blot analysis that confirms successful transfection and strong overexpression of LIMK1 protein in MDA-MB-231 NECTIN2-KD cells compared to KD cells without LIMK1 overexpression. (B) Quantification of NECTIN2 and LIMK1 protein expression normalized to β-actin. (C) Representative wound healing images and (D) quantification of wound closure showing enhanced migration in LIMK1-overexpressing WT and NECTIN2-KD MDA-MB-231 cells. (E) Representative Transwell migration images and (F) the number of migrated cells showing enhanced migration in LIMK1-overexpressing WT and rescue in NECTIN2-KD MDA-MB-231 cells. (G) Representative Transwell invasion images and (H) quantification of invaded cells demonstrating that LIMK1 overexpression partially restores the invasive capacity of NECTIN2-depleted MDA-MB-231 cells. Data are presented as mean ± SEM. Statistical significance was determined using one-way ANOVA followed by Tukey’s multiple comparisons test. For western blot quantification (Fig 5B), n = 3 independent biological replicates. For wound healing (Fig 5D), migration (Fig 5F), and invasion (Fig 5H) assays, n = 3–4 independent biological replicates, each performed in duplicate technical replicates.

Due to the marked reduction in LIMK1, we next examined whether restoring LIMK1 expression could rescue the impaired migratory phenotype of NECTIN2-knockdown MDA-MB-231 cells. We overexpressed LIMK1 in WT and *NECTIN2*-KD MDA-MB-231 cells. Western blot analysis and quantification of band intensities confirmed successful LIMK1 overexpression in WT and NECTIN2-KD cells, while NECTIN2 expression remained suppressed in NECTIN2-KD cells (Fig 5A–B). We performed a wound healing assay to assess the ability of LIMK1-overexpressing MDA-MB-231 *NECTIN2*KD cells to migrate. LIMK1-overexpressing MDA-MB-231 cells exhibited enhanced migration compared to WT cells, confirming LIMK1’s role in promoting migration in breast cancer cells. Interestingly, LIMK1 overexpression also increased the migration ability of *NECTIN2-*KD cells by approximately 50% compared to MDA-MB-231 *NECTIN2-*KD cells, with the rescued migration reaching a similar level to that of WT MDA-MB-231 cells (Fig 5C–D).

Furthermore, Transwell migration assays validated these findings, showing that LIMK1 overexpression increased migration in WT MDA-MB-231 cells and partially rescued the impaired motility of *NECTIN2*-KD cells, restoring the capacity to migrate to a level comparable to that of WT cells (Fig 5E–F). To further determine whether LIMK1 also contributes to the invasive phenotype, Transwell invasion assays were performed. NECTIN2 depletion reduced the number of invaded cells compared to WT cells, while LIMK1 overexpression showed a trend toward increasing the number of invaded cells in both WT and NECTIN2-KD cells; however, the differences did not reach statistical significance (Fig 5G–H). Collectively, these findings suggest that LIMK1 contributes to the migratory phenotype associated with NECTIN2 expression in MDA-MB-231 cells and may also be involved in regulating the invasive phenotype.

### LIMK1 overexpression restores migration and shows a trend toward enhancing invasion in NECTIN2-depleted MDA-MB-231 cells and is associated with partial EMT-like changes

**Fig 6:**
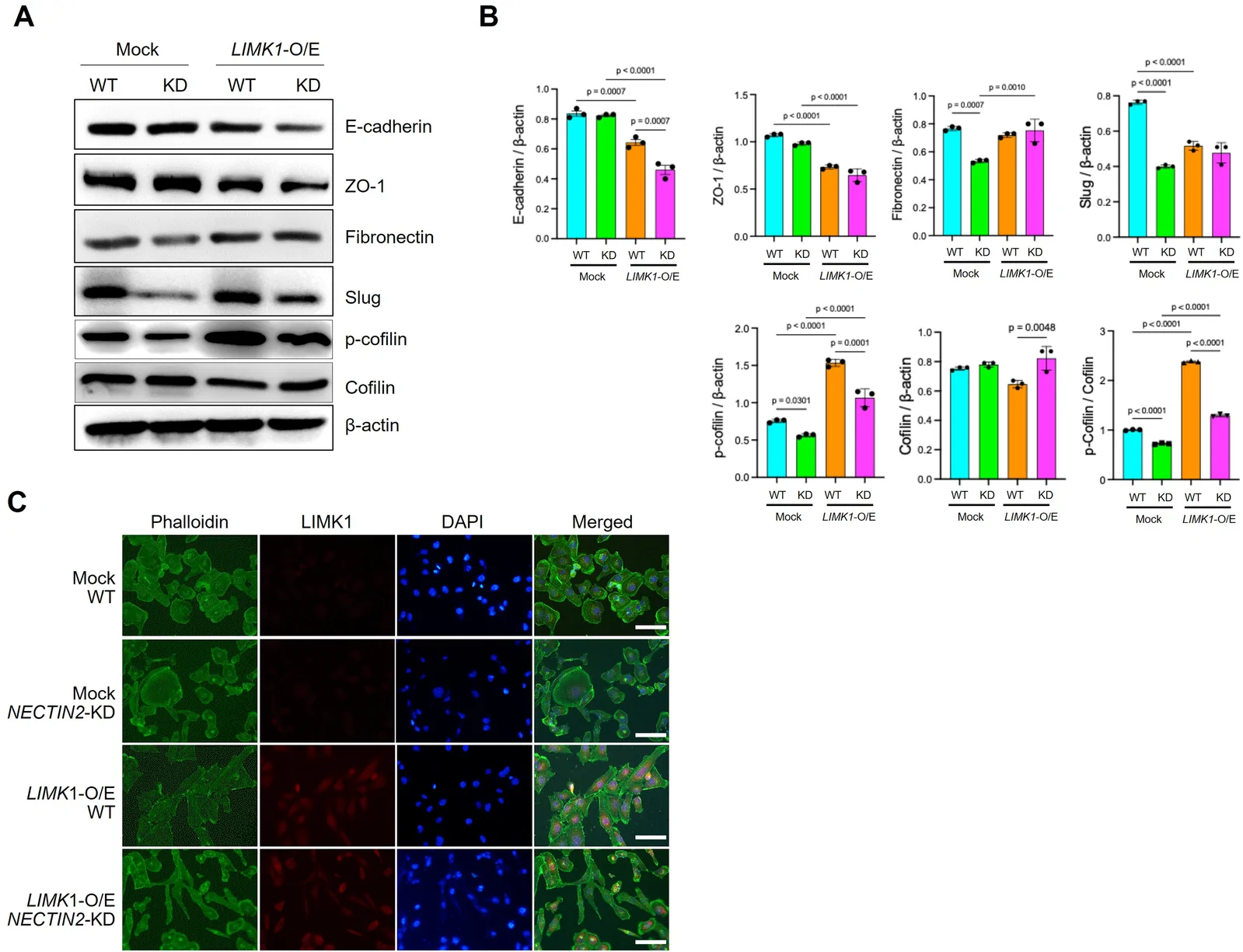
Ectopic LIMK1 expression is associated with EMT-related marker changes and increased cofilin phosphorylation in NECTIN2-depleted MDA-MB-231 cells. (A) Western blot analysis of epithelial markers (E-cadherin and ZO-1), mesenchymal-associated markers (fibronectin and Slug) and cofilin/phospho-cofilin in WT, NECTIN2-KD, and LIMK1-O/E WT and LIMK1 O/E-NECTIN2-KD MDA-MB-231 cells. (B) Quantification of western blot analysis of EMT-related proteins and cofilin phosphorylation. Protein expression levels were normalized to β-actin and presented relative to the control group. Data are presented as mean ± SEM. Statistical significance was determined using one-way ANOVA followed by Tukey’s multiple comparisons test. Quantification was performed from three independent biological replicates (n = 3). (C) Immunofluorescence staining of phalloidin (F-actin), LIMK1 and DAPI showing LIMK1 overexpression, peri-nuclear localization, and more spindle-shaped morphology in LIMK1-overexpressing cells. The representative images were captured using a 20 × objective lens. Scale bar, 100 µm.

To elucidate epithelial and mesenchymal proteins affected by reduced NECTIN2expression and LIMK1 overexpression, we performed western blot analysis to examine epithelial markers (E-cadherin and ZO-1), and mesenchymal markers (fibronectin, Slug), along with cofilin and its phosphorylated form. As shown in Fig 6A and quantified in Fig 6B. Notably, the mesenchymal markers, both fibronectin and Slug were decreased in cells with *NECTIN2-* KD cells compared with control cells (Fig 6A–B), which may be the basis for the impaired migration and invasion abilities observed in these cells. The results also showed decreased expression of E-cadherin and ZO-1 in LIMK1-overexpressing *NECTIN2* KD cells. On the mesenchymal side, a slight increase in fibronectin and a marked upregulation of Slug were observed in LIMK1-overexpressing *NECTIN2*-KD cells compared to the *NECTIN2*-KD cells (Fig 6A–B). These findings indicate that LIMK1 overexpression can partially restore mesenchymal marker expression to support the migratory and invasive abilities of NECTIN2–depleted MDA-MB-231 cells. Moreover, phosphorylated cofilin levels increased in LIMK1-overexpressing WT and *NECTIN2*-KD cells (Fig 6A–B), confirming the downstream activation of LIMK1 signaling.

To further validate LIMK1 overexpression and examine its subcellular localization, immunofluorescence staining was performed for Phalloidin and LIMK1. The results showed that LIMK1 was overexpressed in both LIMK1-overexpressing WT and *NECTIN2*-KD cells, with the predominant LIMK1 localization in the peri-nucleus. Interestingly, LIMK1 overexpression induced a morphological change toward a mesenchymal-like, spindle-shaped morphology compared to WT and *NECTIN2*-KD cells (Fig 6C). These morphological changes were accompanied by alterations in the expression of EMT-related markers, suggesting partial EMT-like changes associated with LIMK1 overexpression.

To further support the functional role of LIMK1 in regulating cell motility, gRNA-mediated LIMK1 depletion was performed in MDA-MB-231 cells. LIMK1 depletion significantly reduced both migration and invasion compared with WT cells (S1 Fig), consistent with the phenotype observed following NECTIN2 depletion. These findings further support the involvement of LIMK1 as a downstream effector of NECTIN2 in regulating the migratory and invasive behavior of MDA-MB-231 cells.

**Fig 7:**
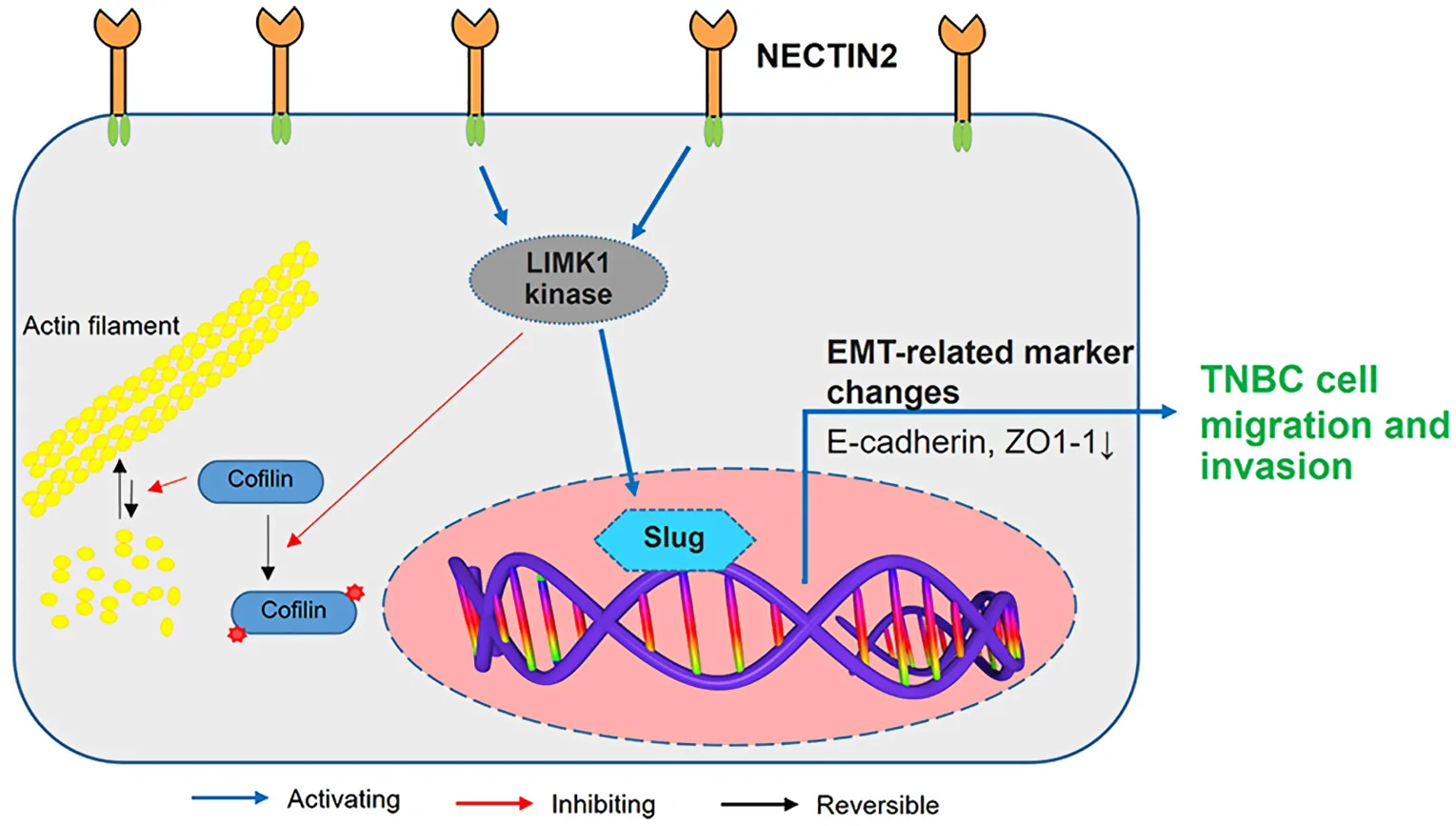
Proposed model of NECTIN2-associated signaling in MDA-MB-231 cells. NECTIN2 may contribute to migratory and invasive phenotypes through modulation of cytoskeletal dynamics. Silencing of NECTIN2 was associated with reduced migratory and invasive capacity, while re-expression of LIMK1 partially restored these phenotypes. Changes in epithelial–mesenchymal transition (EMT)-related markers, including decreased E-cadherin and ZO-1 and increased Slug, were observed in association with LIMK1 re-expression.

Based on the findings of this study, a schematic model summarizing the proposed role of the NECTIN2–LIMK1 axis in regulating migration and invasion in MDA-MB-231 cells is presented in Fig 7.

## Discussion

Triple-negative breast cancer (TNBC) represents the most aggressive subtype of breast cancer, characterized by rapid progression, high metastatic potential, and poor clinical outcomes [4–6]. Unlike other types of breast cancer, TNBC lacks defined therapeutic targets such as hormone receptors or HER2, limiting the effectiveness of current treatments [2,3]. Therefore, identifying novel molecules that regulate the progression and metastasis of TNBC is crucial for the development of new therapeutic strategies.

Our analysis revealed that NECTIN2 expression is significantly upregulated in breast cancer tissues compared to normal controls, with the highest levels observed in TNBC cell lines, particularly MDA-MB-231, at both the mRNA and protein levels. These findings are consistent with previous studies reporting elevated NECTIN2 expression in various malignancies, including breast cancer, and suggest that NECTIN2 may be associated with more aggressive tumor behavior [10,12,20]. Importantly, high NECTIN2 expression was correlated with poorer recurrence-free survival in TNBC patients, supporting the notion that NECTIN2 could serve as a negative prognostic marker in this aggressive subtype, consistent with findings in other types of cancer such as esophageal squamous cell carcinoma [10] and colorectal cancer [8,20] demonstrating that NECTIN2 expression is associated with a poor prognosis and reduced overall survival in lung adenocarcinoma [11]. Given that NECTIN2 belongs to the nectin family of immunoglobulin-like cell adhesion molecules, it likely contributes to cancer progression by modulating cell–cell adhesion and cytoskeletal dynamics, processes that are critical for migration and invasion [10,12]. In particular, while other members of the nectin family, particularly NECTIN4, have been more extensively characterized in breast cancer progression, the functional role of NECTIN2 in TNBC remains relatively underexplored. This gap further supports our initial hypothesis that NECTIN2 may facilitate TNBC cell motility, consistent with our expression data showing that the highest NECTIN2 levels were observed in the highly migratory MDA-MB-231 cell line. Together, these data suggest that NECTIN2 plays a key role in the aggressiveness of TNBC and provide a rationale for further mechanistic studies to explore its contribution to migration, invasion, and metastatic potential.

Therefore, we knocked down NECTIN2 using shRNA in both TNBC cell lines that have the highest expression (MDA‑MB‑231) and moderate expression (MDA‑MB‑468). Interestingly, NECTIN2 depletion suppressed migration and invasion only in MDA‑MB‑231 cells, but not in MDA‑MB‑468 cells, which is consistent with a previous study in esophageal squamous cell carcinoma and lung adenocarcinoma showing that NECTIN2 knockdown effectively suppressed migration and invasion [10,11]. The distinct responses observed between MDA-MB-231 and MDA-MB-468 cells may reflect the intrinsic heterogeneity of TNBC [21]. MDA-MB-231 cells are generally characterized by a more mesenchymal and invasive phenotype, while MDA-MB-468 cells exhibit a more epithelial-like or hybrid epithelial/mesenchymal state [22]. These biological differences, together with the differential expression of NECTIN2 observed between the two cell lines, may influence the contribution of NECTIN2 to migratory and invasive behavior and could explain the context-dependent effects observed in this study.

Because NECTIN2 depletion significantly affected migration and invasion only in MDA-MB-231 cells, but not in MDA-MB-468 cells, subsequent mechanistic analyzes focused on MDA-MB-231 as a representative model of a responsive and highly metastatic TNBC phenotype. NECTIN2 is implicated in the epithelial–mesenchymal transition (EMT), actin cytoskeleton remodeling, and focal adhesion dynamics, all of which are essential for cell motility and invasion [23–25]. In line with these roles, our analysis of gene and protein expression revealed a marked downregulation of LIMK1 following NECTIN2 depletion, suggesting that NECTIN2 may exert its pro-migratory effects through LIMK1-associated cytoskeletal regulation. Moreover, our analysis revealed a moderate and significant positive correlation between NECTIN2 and LIMK1 expression in breast cancer tissues, suggesting that these genes may be functionally connected within tumor-associated regulatory networks. This finding supports our in vitro results and aligns with previous reports indicating that nectin family adhesion molecules can activate Rho-family GTPases and downstream LIMK1, which phosphorylates cofilin to regulate actin filament dynamics and promote lamellipodia and filopodia formation essential for directional migration [26,27]. Although the correlation between NECTIN2 and LIMK1 expression was modest, its statistical significance suggests that this axis may be relevant in aggressive subtypes such as TNBC, warranting further clinical and mechanistic investigation.

Notably, restoration of LIMK1 expression in NECTIN2-depleted MDA-MB-231 cells partially rescued their impaired migratory capacity and was accompanied by increased mesenchymal markers (fibronectin and Slug), decreased epithelial markers (E-cadherin and ZO-1), and elevated phosphorylated cofilin levels, suggesting enhanced actin remodeling and changes associated with EMT. In breast cancer cells, LIMK1 has been shown to enhance metastatic potential; for example, overexpression of LIMK1 in MDA‑MB‑231 cells resulted in increased phospho-cofilin, FAK and paxillin activation, and increased invasive behavior [14]. Conversely, LIMK1 knockdown or pharmacological inhibition reduced breast cancer cell motility by disrupting actin dynamics and EMT-related marker expression [28]. LIMK1 overexpression induced spindle-shaped morphology and nuclear accumulation in MDA-MB-231 cells that are consistent with partial EMT-like changes. This observation agrees with McConnell et al. (2011), who reported that both cytoplasmic and nuclear LIMK1 promote breast cancer progression, with nuclear LIMK1 enhancing transcriptional programs related to motility and invasion. The nuclear localization of LIMK1 may thus contribute to cytoskeletal remodeling and EMT-associated gene expression, promoting a more invasive phenotype in TNBC cells [14].

These findings collectively support a model in which NECTIN2 contributes to the migratory and invasive phenotypes of MDA-MB-231 cells and is associated with LIMK1-related cytoskeletal remodeling and partial EMT-like changes. Given that both NECTIN2 and LIMK1 have been associated with metastatic progression in several cancers [23,29], targeting the NECTIN2–LIMK1 axis may suppress metastases and limit disease progression in aggressive TNBC. As a cell surface adhesion molecule, NECTIN2 also represents a promising target for immunotherapies, offering a precision medicine approach for TNBC patients.

Although we examined several upstream regulators of LIMK1, including RhoA, Rac1, CDC42, and Src, these pathways did not show notable alterations at the mRNA level after NECTIN2 knockdown. However, these findings do not exclude the potential involvement of these signaling pathways, as their activities are frequently regulated at the protein activation or phosphorylation level. Instead, our results suggest that LIMK1 may function as a potential downstream mediator of NECTIN2 in MDA-MB-231 cells. Nevertheless, we cannot exclude the possibility that NECTIN2 regulates LIMK1 through noncanonical or adhesion-dependent mechanisms that were not examined in this study. Future studies will be required to determine the molecular mechanisms linking NECTIN2 to LIMK1 regulation, including investigations of transcriptional and promoter regulation, signaling pathway mapping, and protein stability or degradation mechanisms.

This study offers important insights, but also highlights opportunities for further investigation. Extending these findings to other types of breast cancer would help to determine the broader relevance of the NECTIN2–LIMK1 axis. Given the well-recognized heterogeneity of TNBC, the NECTIN2–LIMK1 signaling axis identified in this study may not be universally conserved across all TNBC subtypes. Therefore, validation in additional TNBC models, patient-derived systems, and clinical specimens will be important to determine the broader applicability and clinical relevance of the NECTIN2–LIMK1 axis. In vivo studies will be valuable to define how NECTIN2–LIMK1 signaling influences tumor growth, metastatic progression, and interactions within the tumor microenvironment, thereby strengthening the translational significance of this pathway. One limitation of the present study is that the functional characterization of NECTIN2 was based on a single shRNA construct. While complementary LIMK1 rescue and depletion experiments provided additional functional support for the proposed pathway, additional validation using an independent shRNA sequence or an alternative gene-silencing approach would further strengthen the specificity of the NECTIN2 knockdown findings.

In conclusion, NECTIN2 may contribute to migratory and invasive phenotypes in MDA-MB-231 cells, potentially through LIMK1-associated regulation of cytoskeletal dynamics and partial EMT-like changes. NECTIN2 depletion was associated with reduced LIMK1 expression and impaired migration and invasion, while re-expression of LIMK1 partially restored migratory capacity and was accompanied by changes in EMT-related markers, including decreased epithelial markers (E-cadherin and ZO-1) and increased mesenchymal regulators such as Slug and fibronectin. These findings suggest a model in which the NECTIN2–LIMK1 axis is linked to metastatic traits in MDA-MB-231 cells. However, this relationship appears to be dependent on context and the precise molecular mechanisms require further investigation.

## Supporting information

S1 Table Primer sequences and PCR conditions used for qPCR analysis. (DOCX)

S1 Fig gRNA-mediated LIMK1 (Addgene #76047) depletion suppresses migration and invasion in MDA-MB-231 cells. (A) Western blot confirming LIMK1 depletion in LIMK1-gRNA MDA-MB-231 cells compared with WT cells. β-actin was used as a loading control. (B) Representative images of migrated cells in the transwell migration assay. (C) Quantification of migrated cells. (D) Representative images of invaded cells in the Matrigel invasion assay. (E) Quantification of invaded cells. Data from the migration and invasion assays are presented as mean ± SD from three technical replicates. Technical replicates were averaged before statistical analysis. Statistical significance was determined using the Mann–Whitney U test. P < 0.05.
(DOCX)

S1 File Raw images. (PDF)

## Abbreviations

BC: breast cancer
TNBC: triple-negative breast cancer
NECTIN2: Nectin cell adhesion molecule-2
shRNA: short hairpin RNA
LIMK1: LIM kinase 1
EMT: epithelial–mesenchymal transition.
