# Correction: NECTIN2 depletion impairs migration and invasion in MDA-MB-231 breast cancer cells through LIMK1-associated cytoskeletal remodeling

**DOI:** 10.1371/journal.pone.0359312

**Published:** 2026-09-25

**Authors:** Phatchanat Klaihmon, Pattaratorn Muangtate, Sudarat Thongphayong, Puretat Saetan, Supasorn Chanthateyanonth, Chanitra Thuwajit, Surapol Issaragrisil, Phatchariya Phannasil

The seventh author’s name is spelled incorrectly. The correct name is: Surapol Issaragrisil.
