# Supplementary material for: NECTIN2 depletion impairs migration and invasion in MDA-MB-231 breast cancer cells through LIMK1-associated cytoskeletal remodeling
Source: PLoS One. 2026 Aug 28;21(8):e0356726. doi: 10.1371/journal.pone.0356726 (PMC13524328; doi:10.1371/journal.pone.0356726)
Supplement: Supplementary file 1 [file pone.0356726.s003.pdf]

Uncropped WB

Nectin-2 in TNBC

15-04-2026

Fig 1E

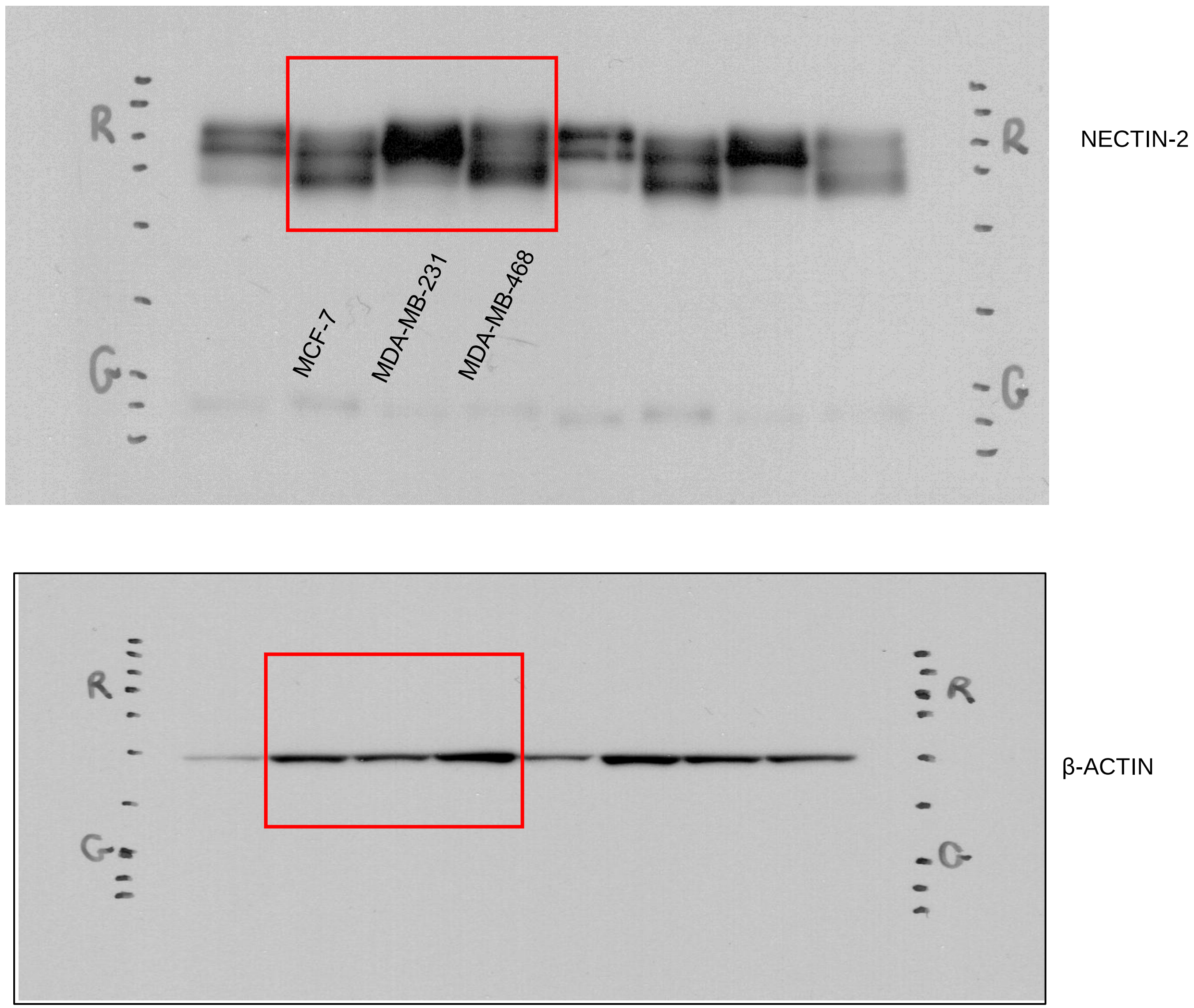

Fig 2B

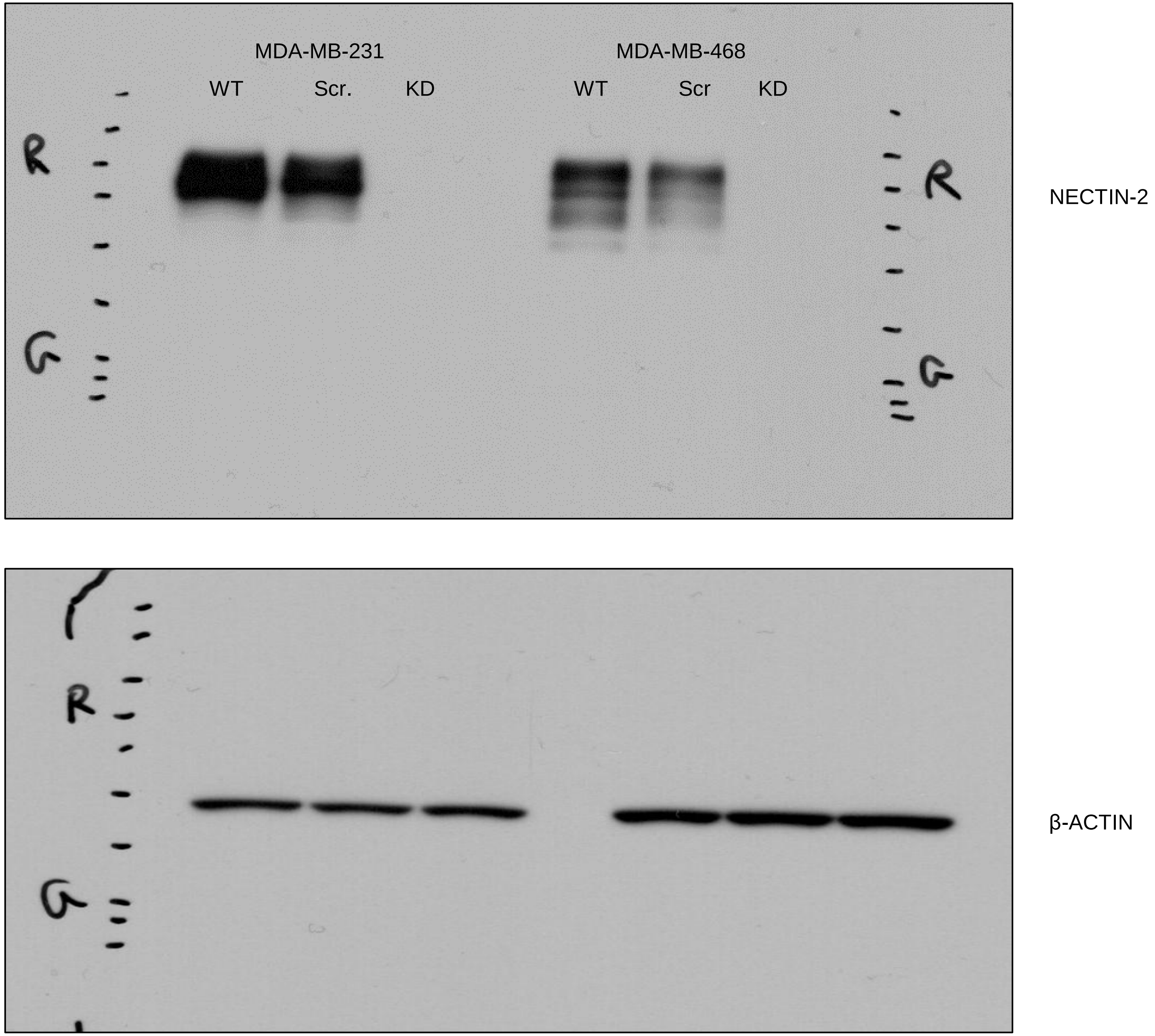

Fig 4C

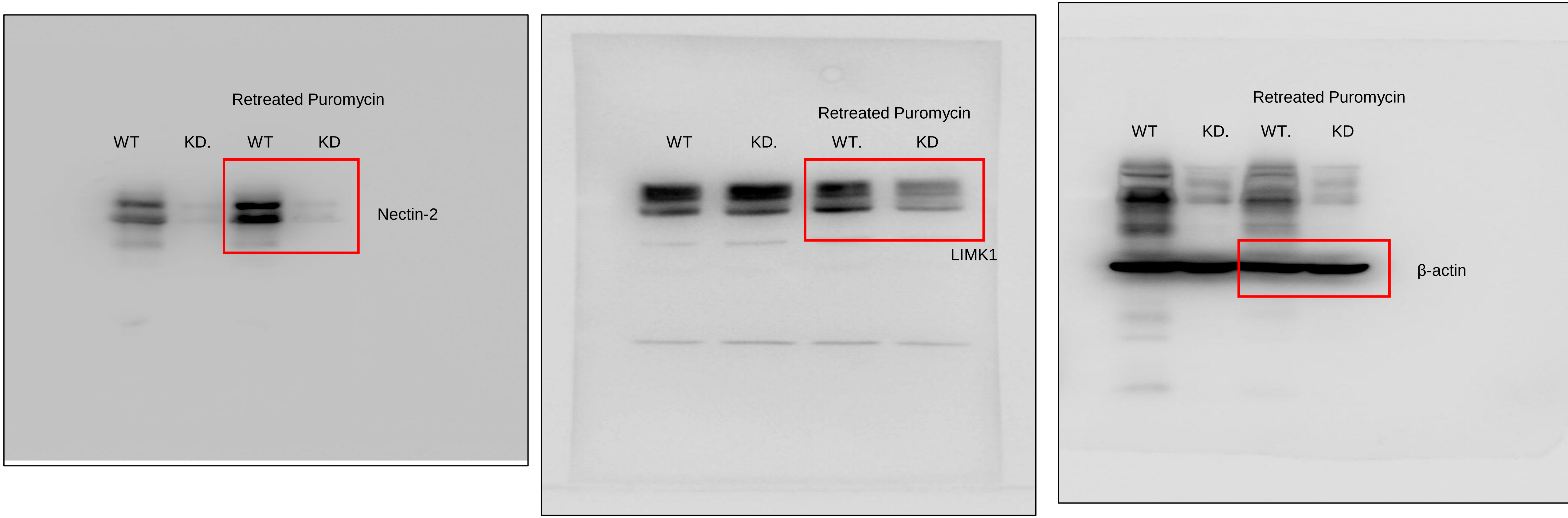
Retreated Puromycin
WT
KD.
WT
KD
Nectin-2
Retreated Puromycin
WT
KD.
WT.
KD
LIMK1
Retreated Puromycin
WT
KD.
WT.
KD
β-actin

Fig 5A

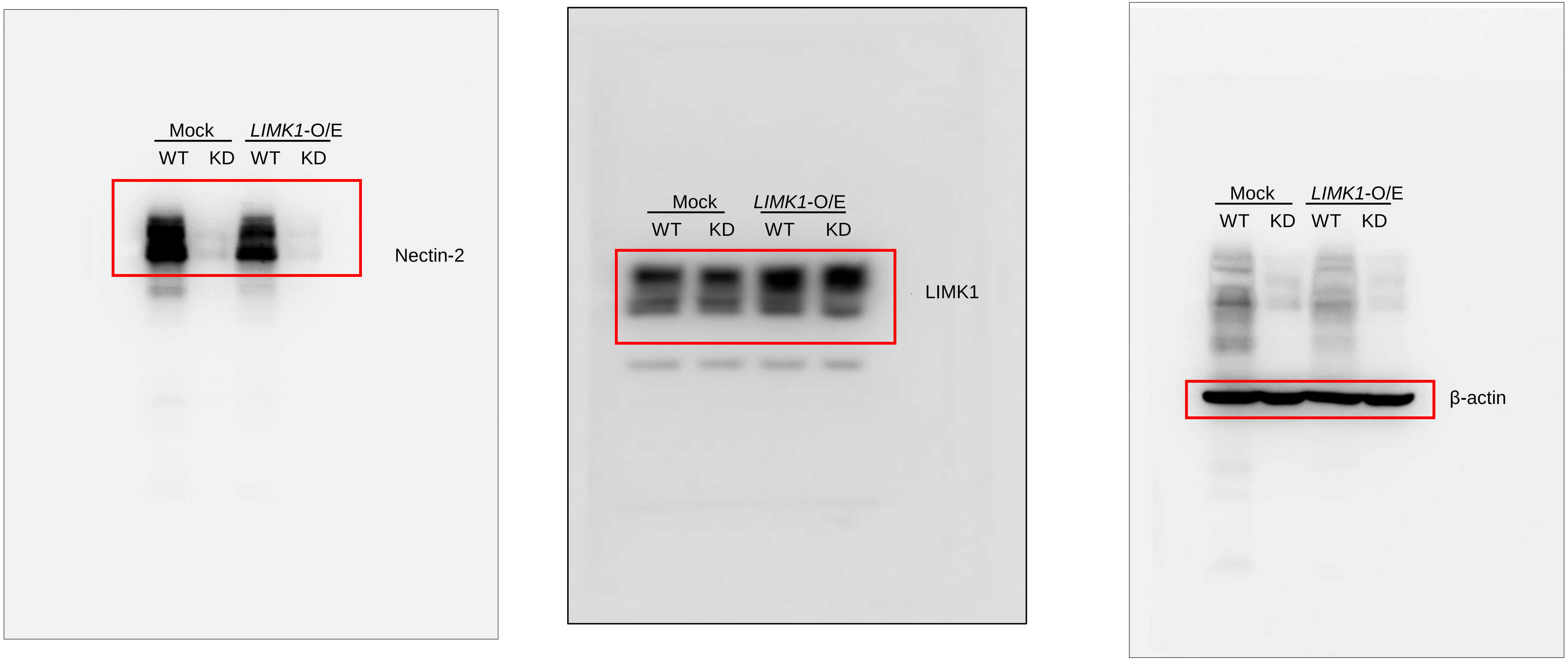
Mock
LIMK1-O/E
WT KD WT KD
Nectin-2
Mock
LIMK1-O/E
WT KD WT KD
LIMK1
Mock
LIMK1-O/E
WT KD WT KD
β-actin

Fig 6A

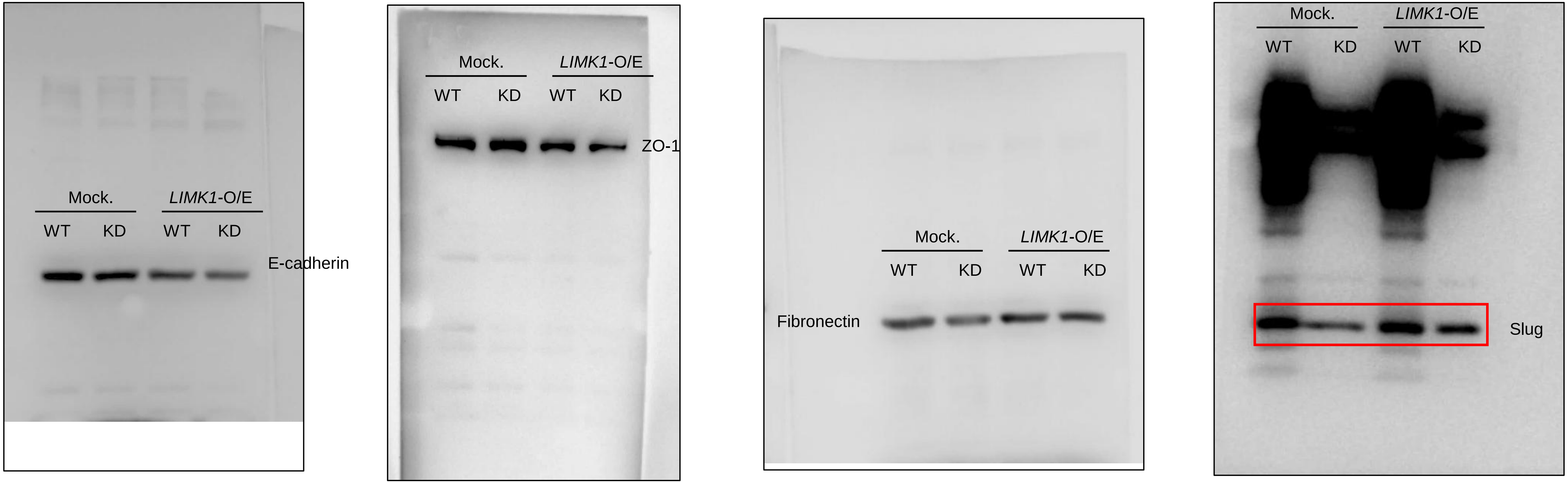
Mock.
LIMK1-O/E
WT
KD
WT
KD
E-cadherin
Mock.
LIMK1-O/E
WT
KD
WT
KD
ZO-1
Mock.
LIMK1-O/E
WT
KD
WT
KD
Fibronectin
Mock.
LIMK1-O/E
WT
KD
WT
KD
Slug

Fig 6A

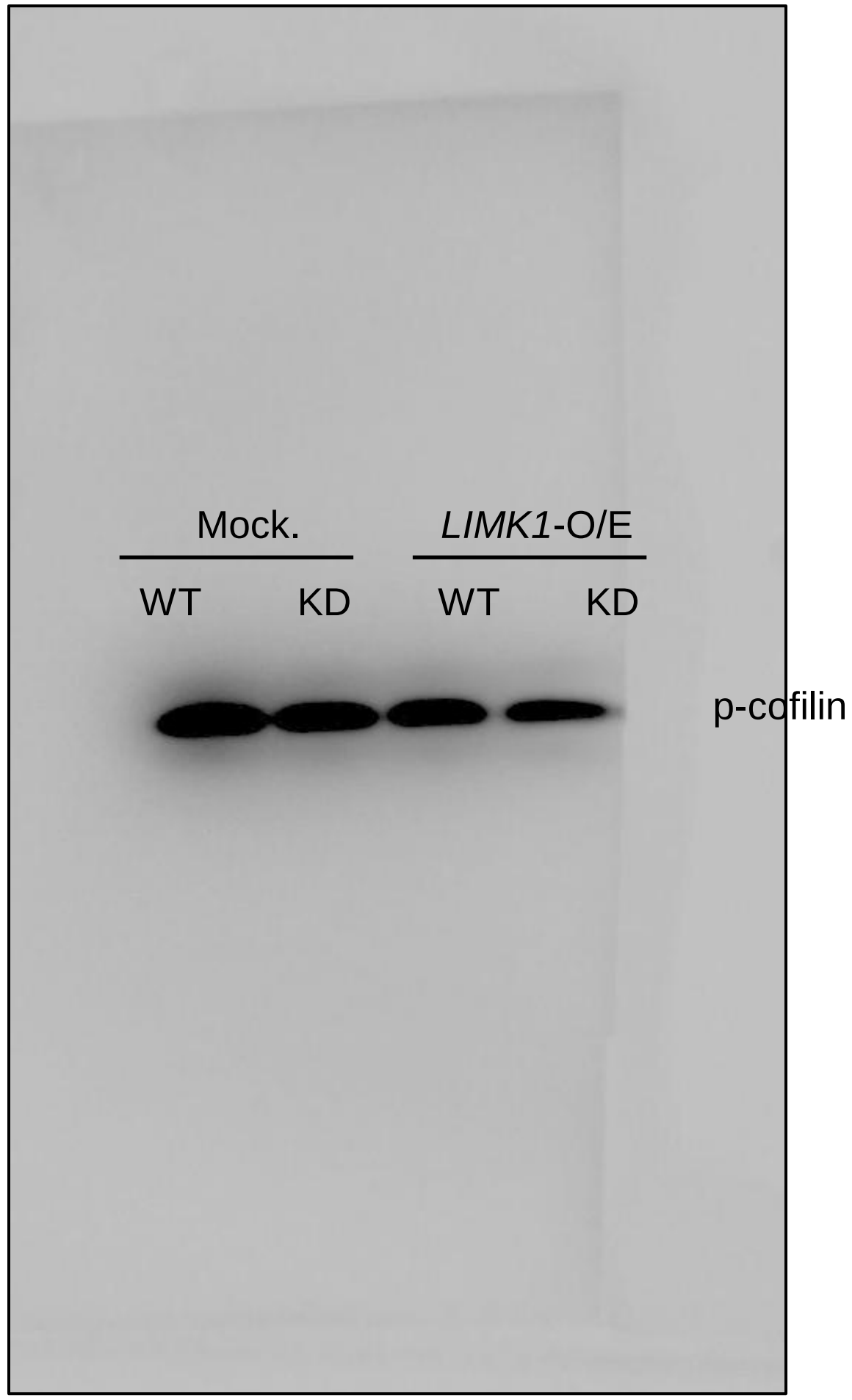

Mock.
LIMK1-O/E
WT
KD
WT
KD
p-cofilin

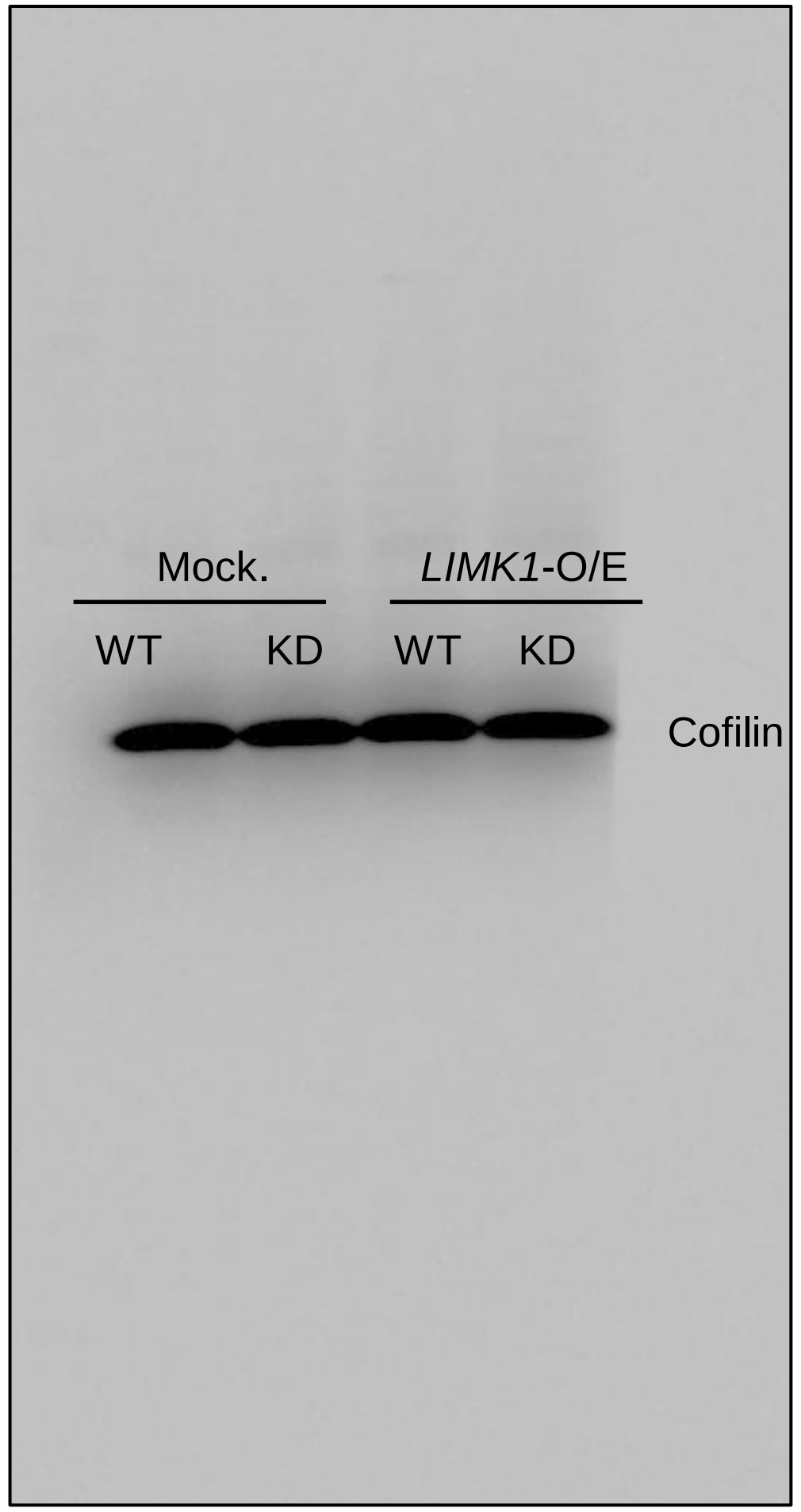

Mock.
LIMK1-O/E
WT
KD
WT
KD
Cofilin

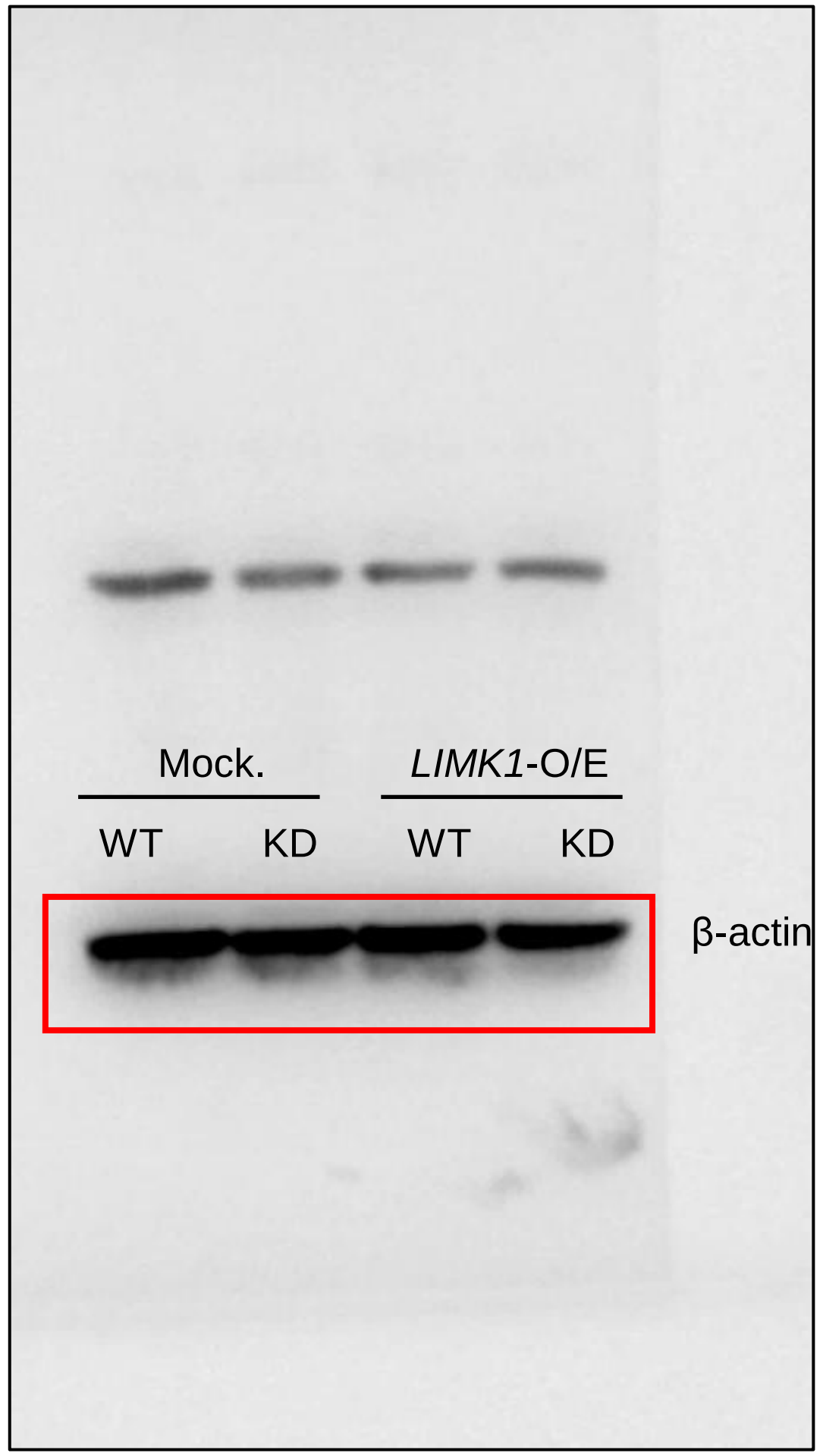

Mock.
LIMK1-O/E
WT
KD
WT
KD
β-actin
