# Supplementary material for: NECTIN2 depletion impairs migration and invasion in MDA-MB-231 breast cancer cells through LIMK1-associated cytoskeletal remodeling
Source: PLoS One. 2026 Aug 28;21(8):e0356726. doi: 10.1371/journal.pone.0356726 (PMC13524328; doi:10.1371/journal.pone.0356726)
Supplement: Supplementary file 2 [file pone.0356726.s001.docx]

**Supplementary Table S1:** Primer sequences and PCR conditions used for qPCR analysis

| **No.** | **Gene/ Primer name** | **Sequence 5’ – 3’** | **Ta (°C)** | **Product size (bp)** |
| --- | --- | --- | --- | --- |
| 1 | NECTIN2_F | ACTGATGCCACCCTGAGCTG | 62.4 | 172 |
| 2 | NECTIN2_R | CATTGGTGACTGTGCAGACGAAG |  |  |
| 3 | ACTB_F | TCACCAACTGGGACGACATGG | 62 | 152 |
| 4 | ACTB_R | GGTCTCAAACATGATCTGGGTC |  |  |
| 5 | SRC_F | TGGCTTCTGCTGTTGACTGG | 59 | 109 |
| 6 | SRC_R | GAGGATGGTCAGGTTGTGCT |  |  |
| 7 | CDC42_F | ACGACCGCTGAGTTATCCAC | 58 | 126 |
| 8 | CDC42_R | GGAGTCTTTGGACAGTGGTGA |  |  |
| 9 | RAC1_F | TCCGCAAACAGATGTGTTCTTA | 58 | 111 |
| 10 | RAC1_R | GGAGTGTTGGGACAGTGGTG |  |  |
| 11 | RhoA_F | TCGTTAGTCCACGGTCTGGT | 57 | 118 |
| 12 | RhoA_R | CAGCCATTGCTCAGGCAAC |  |  |
| 13 | VAV2_F | TCCGGTCCATAGTCAACCAC | 55 | 178 |
| 14 | VAV2_R | TTCCCGTGAGACTTCTTGAC |  |  |
| 15 | RAP1A_F | ATGGCATTGAGTAGATCGTCA | 57 | 198 |
| 16 | RAP1A_R | GCATACACTGTTGGCAATCG |  |  |
| 17 | TIAM1_F | GCTGAACACGTCAGGTGGTA | 59 | 150 |
| 18 | TIAM1_R | CCCGTGGAGTCCAGATGAAG |  |  |
| 19 | AKAP3_F | TCCTGTTCCCTGAAAATTCCC | 58 | 138 |
| 20 | AKAP3_R | AGTGAACCACGGAAGACTCC |  |  |
| 21 | MMP-7_F | CATGATTGGCTTTGCGCGAG | 56 | 144 |
| 22 | MMP-7_R | CTACCATCCGTCCAGCGTTC |  |  |
| 23 | MEKK-1_F | TGATCACAAGCAGGGGGTTC | 57 | 194 |
| 24 | MEKK-1_R | CACAATGAGCCACAACTGCC |  |  |
| 25 | MAP2K4_F | ACAGGAGTTCAAAACCCACACA | 58 | 108 |
| 26 | MAP2K4_R | GTCCTCTGCAGTGAAATCCCA |  |  |
| 27 | JNK_F | GGTCTTGCAGCCTTACAGTC | 58 | 121 |
| 28 | JNK_R | GCTTTTGTCAGGCACAGCAC |  |  |
| 29 | SPIRE2_F | AGGGAACAGGGTGGGTTTTC | 58 | 170 |
| 30 | SPIRE2_R | CAGCTGGGAGCCAAGATACC |  |  |
| 31 | LIMK1_F | GCTTCCTCCAGAGGGCTAAG | 58 | 190 |
| 32 | LIMK1_R | CACTCGGGGTGGTACTTCAG |  |  |
| 33 | LIMK2_F | GTTCCCACAGATTGTCCCCC | 62 | 191 |
| 34 | LIMK2_R | AGGTTGAGGTGCTTCCGTAG |  |  |

Note: forward primer (F), reverse primer (R), Annealing temperature (Ta)
