# Supplementary material for: NECTIN2 depletion impairs migration and invasion in MDA-MB-231 breast cancer cells through LIMK1-associated cytoskeletal remodeling
Source: PLoS One. 2026 Aug 28;21(8):e0356726. doi: 10.1371/journal.pone.0356726 (PMC13524328; doi:10.1371/journal.pone.0356726)
Supplement: Supplementary file 3 [file pone.0356726.s002.docx]

**Supplementary Figure S1**

**Supplementary Figure S1**. gRNA-mediated LIMK1 (Addgene #76047) depletion suppresses migration and invasion in MDA-MB-231 cells. (A) Western blot confirming LIMK1 depletion in LIMK1-gRNA MDA-MB-231 cells compared with WT cells. β-actin was used as a loading control. (B) Representative images of migrated cells in the transwell migration assay. (C) Quantification of migrated cells. (D) Representative images of invaded cells in the Matrigel invasion assay. (E) Quantification of invaded cells. Data from the migration and invasion assays are presented as mean ± SD from three technical replicates. Technical replicates were averaged before statistical analysis. Statistical significance was determined using the Mann–Whitney U test. P < 0.05.
